# A Rare Case of Intraoperative Anaphylaxis Secondary to Central Venous Catheter Placement: A Case Report

**DOI:** 10.1155/cria/1310392

**Published:** 2025-02-11

**Authors:** Yusuf Ali, Andres Gonzalez, John Pallan, Emily Anne Smith Bergbower

**Affiliations:** Department of Anesthesiology, University of Maryland Medical Center, Baltimore, Maryland, USA

**Keywords:** anaphylaxis, anesthesiology, chlorhexidine, impregnated central line, intraoperative resuscitation, perioperative medicine

## Abstract

Central venous catheters (CVCs) are often used intraoperatively for large volume resuscitation and the administration of vasoactive medications. Many of these catheters are impregnated with antimicrobials to prevent infection but independent reports of anaphylaxis directly following catheter placement have been increasing. Here, we report the case of a severe episode of anaphylaxis attributed to a chlorhexidine-coated CVC in a middle-aged man who presented for an L5-S1 Anterior Lumbar Interbody Fusion (ALIF) revision due to hardware failure.

## 1. Introduction

Central venous catheters (CVCs) play a critical role in venous access for perioperative and ICU physicians. These specialized lines are impregnated with various bactericidal compounds to reduce catheter-related bloodstream infections. Chlorhexidine, a synthetic bisbiguanide, is a common bactericidal agent found in the composition of several CVC products across more than one brand [1, 2]. It also serves as a skin disinfectant and cleansing agent applied to the body prior to surgery. Chlorhexidine-impregnated CVCs were first introduced into clinical practice circa 1990 and its bactericidal function occurs via disruption of the microbial cell membrane and precipitation of intracellular contents [1].

The ubiquity of chlorohexidine increases the risk of sensitization in the general population [3]. This increased sensitization may have implications in the perioperative setting in which chlorhexidine related reactions occur. The true incidence of such events is unknown, likely due to both underreporting and difficulty in establishing a causal relationship between exposure and reaction. The first indexed case report of anaphylaxis secondary to a chlorhexidine-impregnated CVC was published in 1997 and an FDA warning has existed since 1998 [4].

### 1.1. Disclosure

This patient provided written Health Insurance Portability and Accountability Act (HIPAA) authorization and consent for publication of this case report.

## 2. Case Presentation

A 57-year-old man with no significant past medical history or known allergies was initially evaluated outpatient by orthopedic surgery for operative management of lumbar spinal stenosis and L5-S1 spondylolisthesis. He subsequently underwent L5-S1 Anterior Lumbar Interbody Fusion (ALIF) but developed worsening back pain in the year following surgery with plain films demonstrating L5-S1 anterior hardware failure and significant graft protrusion out of the L5-S1 disc space. Follow-up CT imaging confirmed hardware failure with tenting of the intraabdominal vascular structures and the patient was scheduled for urgent hardware removal with revision of the L5-S1 ALIF as well as posterior decompression and fusion.

On the day of surgery, the patient presented to the preoperative waiting area from home. He was afebrile with a heart rate (HR) of 84 beats per minute (bpm), BP of 142/78 mmHg, and an oxygen saturation (SpO_2_) of 98% on room air. The patient reported good functional capacity with metabolic equivalents (METS) > 4. Given his overall health and functional status, no labs were collected on the day of surgery except a type and screen. Upon arrival to the OR, the patient was preoxygenated by face mask. General anesthesia was induced with 150 mg of propofol, 2 mg of midazolam, and 100 mcg of fentanyl. He was subsequently paralyzed with 100 mg of rocuronium. Intubation was achieved in one attempt with an 8.0 endotracheal tube. He had two large-bore peripheral IVs in place as well as a radial arterial line for close hemodynamic monitoring.

The arterial line was placed prior to CVC insertion. Subsequently, a double-lumen access catheter (MAC) was placed in the right internal jugular vein after the skin was prepped with 2% chlorhexidine. The catheter underwent manometry testing indicating venous placement and the patient's vital signs remained unchanged during the procedure. Approximately two minutes after line placement, the patient developed profound hemodynamic instability with a decrease in mean arterial pressure (MAP) from 78 (102/65) to 26 (30/24) and an increase in HR from 75 to 168.

**Figure 1:**
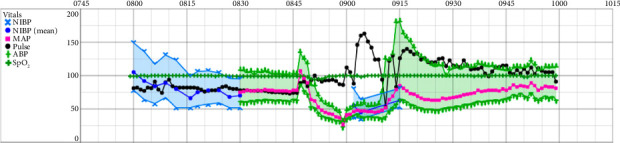
Intraoperative anesthetic record demonstrating arterial blood pressure (ABP), heart rate (HR), and pulse oxygen saturation (SpO_2_) changes during the episode of anaphylaxis.

For the purpose of resuscitation, the patient received 4 L of crystalloid fluid as well as 2000 mcg of phenylephrine, 50 mg of ephedrine, and 2 g of calcium chloride given in divided doses. Hemodynamically, no response was observed to these interventions and the patient remained in refractory shock. Subsequently, all anesthetics were turned off and six 100 mcg boluses of epinephrine were given, followed by initiation of an epinephrine infusion. The patient's blood pressure stabilized after treatment with epinephrine, approximately 15 min after the initial decompensation (Figure 1). At this time, an arterial blood gas (ABG) revealed a pH of 7.26, partial pressure of carbon dioxide (pCO2) of 48, partial pressure of oxygen (pO2) of 316, bicarbonate (HCO_3_) of 21, base excess of −5.6, and lactate of 2.1. Laboratory values were significant for a potassium of 2.7, calcium of 1.30, and a sodium of 138. EtCO_2_ ranged from 30 to 40 throughout the entire episode.

**Figure 2:**
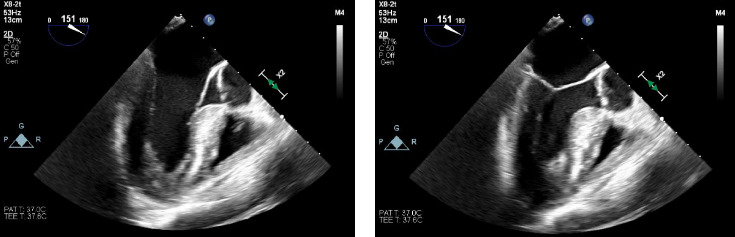
Intraoperative TEE. Mild SAM noted without significant LVOT obstruction. (a) Ventricular diastole. (b) Ventricular systole.

Once the patient stabilized, CVC placement was confirmed fluoroscopically in the OR. An intraoperative transesophageal echocardiogram (TEE) demonstrated mild systolic anterior motion (SAM) without significant left ventricular outflow obstruction (LVOT), preserved LV and RV function, normal ventricular wall motion, and no evidence of a pericardial effusion (Figure 2). At this time, the patient was noted to have significant facial edema and lip swelling. The case was aborted due to the concern for anaphylaxis, and a tryptase level was drawn. The patient was taken to the surgical ICU for postoperative monitoring where the intraoperatively drawn tryptase level resulted confirming anaphylaxis. The CVC was removed and the epinephrine infusion was discontinued soon afterward. He was discharged 2 days later with the planned ALIF rescheduled for 1 week in the future.

### 2.1. Follow-Up

Skin prick testing for both chlorhexidine (CHG) and nondepolarizing neuromuscular blockers (NMBAs) was recommended at discharge, as these two agents were strongly suspected to be the potential underlying cause of the described anaphylactic episode. However, skin prick testing was delayed due to the patient's unstable spine lesion and risk of permanent neurological damage. Surgery was rescheduled for 1 week after discharge from the hospital for this anaphylactic episode. The use of CHG antiseptic preparation and CHG-impregnated CVCs were avoided in the rescheduled case. In addition, succinylcholine was used in the subsequent operation in place of rocuronium. As an additional precaution, the patient was given 50 mg of IV diphenhydramine and 125 mg IV of methylprednisolone shortly after induction. No CVC was placed and the patient tolerated the surgery well. Subsequent skin prick testing by an allergist revealed a chlorhexidine allergy.

## 3. Discussion

Anaphylaxis during anesthesia is rare, with an incidence of 1 in 5000–25,000 cases [5]. Initially, our differential diagnosis was very broad to evaluate the different causes of severe, vasopressor-refractory hypotension. Hypovolemia was quickly ruled out as the patient received 4 L of crystalloid without improvement in hemodynamics and the surgery had not yet begun. Hypoxia/hypoxemia and electrolyte abnormalities were also ruled out as stat labs, including an ABG, revealed adequate oxygenation with a pO_2_ of 316 while on 100% FiO_2_ and normal electrolyte values. While acidosis was on the differential, we confirmed that only a mild lactic acidosis was present, likely the result of malperfusion in the setting of hemodynamic collapse.

Intraoperative fluoroscopy confirmed correct placement of the CVC, without evidence of pneumothorax. The intraoperative TEE ruled out cardiac tamponade and acute coronary syndrome. In addition, the TEE did not demonstrate signs of pulmonary thrombosis including an embolism or right heart strain. The SAM seen on TEE was likely a result of the epinephrine infusion that was initiated in the OR as opposed to a left ventricular outflow obstruction that led to hemodynamic collapse.

Anaphylaxis can be an IgE-mediated or IgE-independent reaction, resulting in the release of chemical mediators such as histamine and tryptase from mast cell activation [6]. The release of these chemical mediators is what ultimately leads to the refractory hypotension and hemodynamic collapse. In this case, the patient had physical exam findings concerning for anaphylaxis and a rapidly drawn tryptase level to support the diagnosis. Obtaining a tryptase level is the key for a diagnosis, but levels begin to normalize after 3–4 hours, so samples should be collected as soon as possible, ideally in the first 2 hours per EAACI guidelines, if anaphylaxis is suspected [7]. In this particular case, the chlorhexidine-coated central line seemed the most likely culprit based on the timeline of events.

Chlorhexidine-impregnated CVCs have become commonplace in the perioperative environment in order to reduce the frequency of catheter-related infections. Although this approach has succeeded in its initial purpose, it is critical for the perioperative physician to be aware of what these catheters are impregnated with chemically and to consider that patients may react to these compounds. Chlorhexidine has become so ubiquitous due to various cleaning products and even over the counter steroid creams that sensitization is likely increasing [3, 8]. These reactions can be delayed or immediate, and an initial delayed reaction can be overlooked prior to severe decompensation on a second exposure. Several adjacent and potential examples of this exist in the literature specific to central lines [9–11]. This report highlights the risk antimicrobial adjuncts in commercially available CVCs may bring to patients who have had prior exposures or documented reactions.

## Data Availability

Data sharing is not applicable as no new data was generated.
